# Diphtheria's Dual Threat: Amplifying Awareness of Cardiac Complications for Enhanced Intervention

**DOI:** 10.7759/cureus.56093

**Published:** 2024-03-13

**Authors:** Saadia Ilyas, Zaland A Yousafzai, Imran Khan, Qazi Kamran Amin, Muhammad Bilal

**Affiliations:** 1 Pediatric Cardiology, Lady Reading Hospital, Peshawar, PAK; 2 Medicine, Rehman Medical Institute, Peshawar, PAK; 3 Interventional Cardiology/Electrophysiology, Lady Reading Hospital, Peshawar, PAK; 4 General Medicine, Rehman Medical Institute, Peshawar, PAK; 5 Cardiology, Lady Reading Hospital, Peshawar, PAK

**Keywords:** diphtheria, pediatric cardiology, pakistan, complications, myocarditis

## Abstract

Even though immunization can prevent illness, diphtheria, which is caused by toxic strains of Corynebacterium diphtheriae, remains a serious public health risk. Although the worldwide incidence has declined, it still poses a serious hazard in developing countries, such as Pakistan, where new data suggest an increase in cases. A significant proportion of patients with respiratory diphtheria experience cardiac complications, specifically myocarditis, which carries a high death risk of 50% to 75%. The diphtheria toxin's affinity for cardiac tissues is the cause of these consequences, which include arrhythmias and myocardial dysfunction. Recent studies from Lady Reading Hospital in Peshawar show the seriousness of the situation, with 73 patients presenting with cardiac complications in just one year, resulting in a devastating fatality rate despite early management. This highlights the pressing need for increased awareness and all-encompassing immunization campaigns, particularly for children who have received insufficient vaccinations. Timely vaccination and booster doses are critical for reducing myocarditis-related mortality, mandating prioritizing immunization efforts to defend susceptible populations globally.

## Editorial

Caused by toxigenic strains of Corynebacterium diphtheria, diphtheria is a multisystem infectious disease that can be prevented with vaccination but has the potential to be lethal. Although its frequency has decreased, it continues to be rampant in many developing nations, such as Pakistan. The WHO reports that starting in 2015, there has been an increase in the number of cases of diphtheria each year [1]. In the first three quarters of 2022, there were 342 recorded cases of diphtheria as disclosed by National Health Services Regulation and Coordination, Government of Pakistan [2]. The province of Khyber Pakhtunkhwa (KPK) has recently witnessed a surge in the number of diphtheria cases, According to the KP Health Department, there are presently 259 suspected cases across the province, and it is estimated that as much as 80% of the patients are young children [3]. 

Cardiac complications, specifically Myocarditis, occur in approximately 10-25% of patients with respiratory diphtheria. It is a complication associated with a high mortality rate. which varies from 50% to 75%. Cardiac manifestations can be variable and include myocardial dysfunction, Rhythm abnormalities including sinus tachycardia, T wave inversion, ST segment depression, right bundle branch block, multiple atrial ectopics (3.3%), and heart blocks requiring pacemaker placement [4]. The diphtheria toxin has an affinity for cardiac myocytes and the cardiac conduction system, which makes cardiac problems frequent and well-documented. The breakdown of actin filaments induced by the diphtheria toxin results in myocarditis and impairs contractile functioning. In individuals who recover, damaged cardiomyocytes are gradually replaced with fibrotic tissue, which can contribute to long-term cardiac sequelae. Cardiac problems can also arise in persons infected with nontoxigenic strains of Corynebacterium (C.) diphtheriae. Cardiac involvement in diphtheria is variable but most typically characterized by myocardial dysfunction and arrhythmias and, occasionally, pericarditis and endocarditis. The existence of a bull neck and the amount of pharyngeal pseudomembrane upon admission are potential risk factors for cardiomyopathy [5].

Despite significant advances in medical treatment, treating these diseases is still difficult, particularly in areas that lack adequate infrastructure and resources for healthcare. Alarmingly, recent data from the pediatric cardiology department of Lady Reading Hospital in Peshawar, the largest hospital in the region, show the seriousness of the issue in question. Over a period of a single year, from January 2023 to January 2024, a total of 73 patients presented with cardiac complications as a consequence of diphtheria. Among these cases, 31 victims unfortunately expired due to the condition despite receiving what was judged prompt intervention and treatment. This grim reality not only underlines the aggressiveness of the disease but also underscores the urgent need for heightened awareness, robust preventive measures, and more effective management tactics in such circumstances. In conclusion, the persistence of myocarditis among under-vaccinated children, particularly in underprivileged regions, remains an imminent issue due to its staggering mortality rates, emphasizing the need for comprehensive immunization efforts. Given the danger of myocarditis death once it develops, vaccination in conjunction with timely booster doses is especially important for the pediatric population. Hence, it is vital to prioritize immunization efforts to reduce the occurrence of diphtheria and its associated cardiac complications, eventually defending the health and well-being of vulnerable populations globally.
